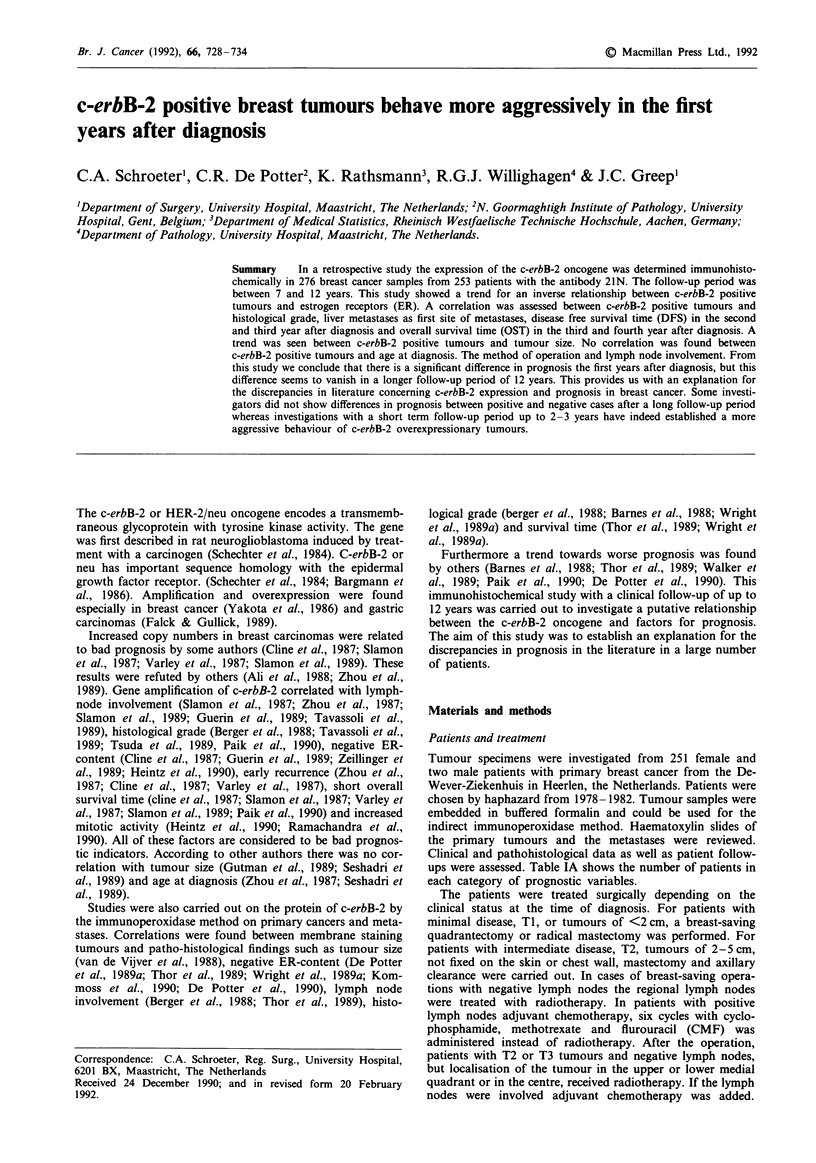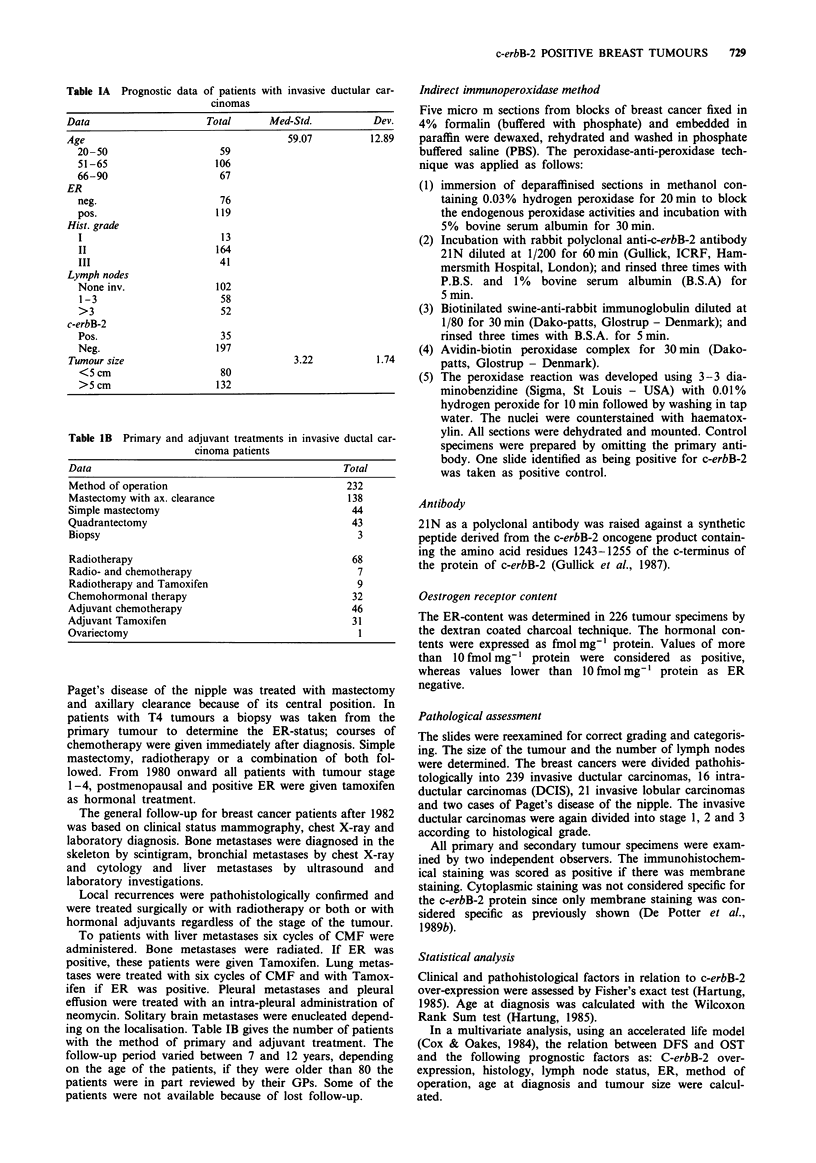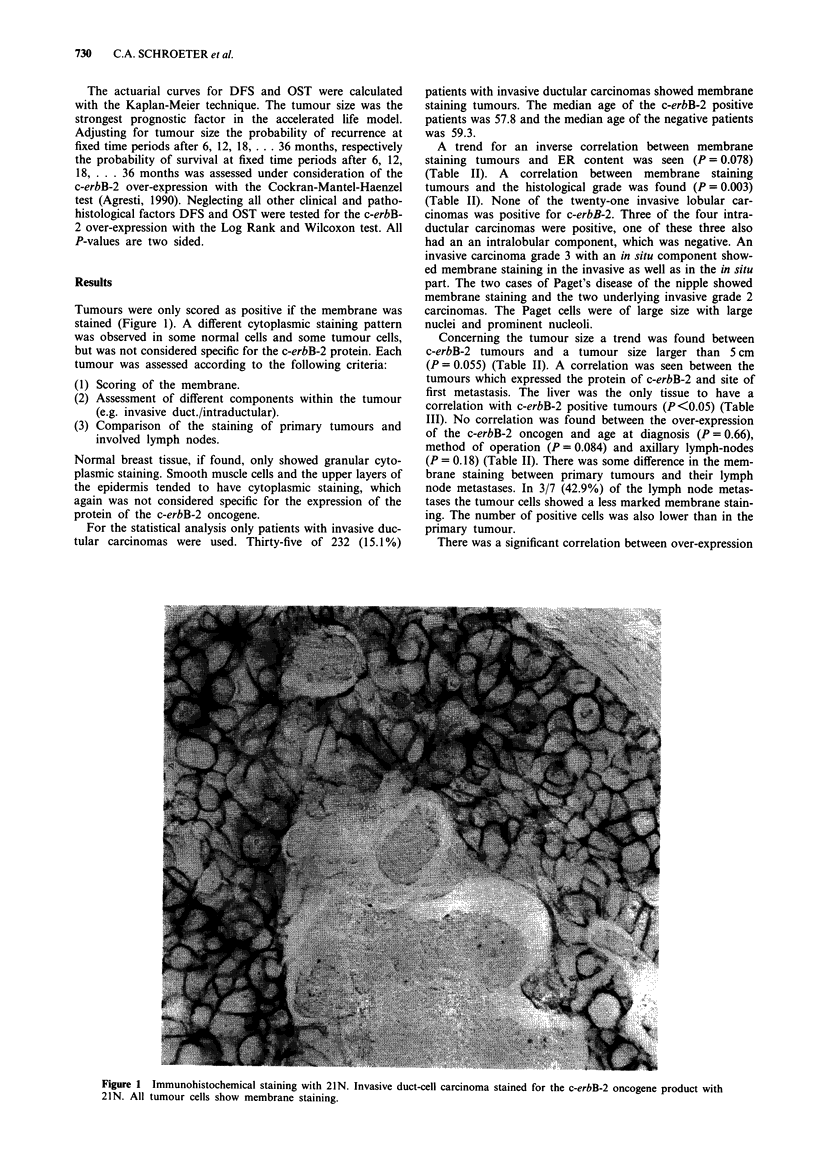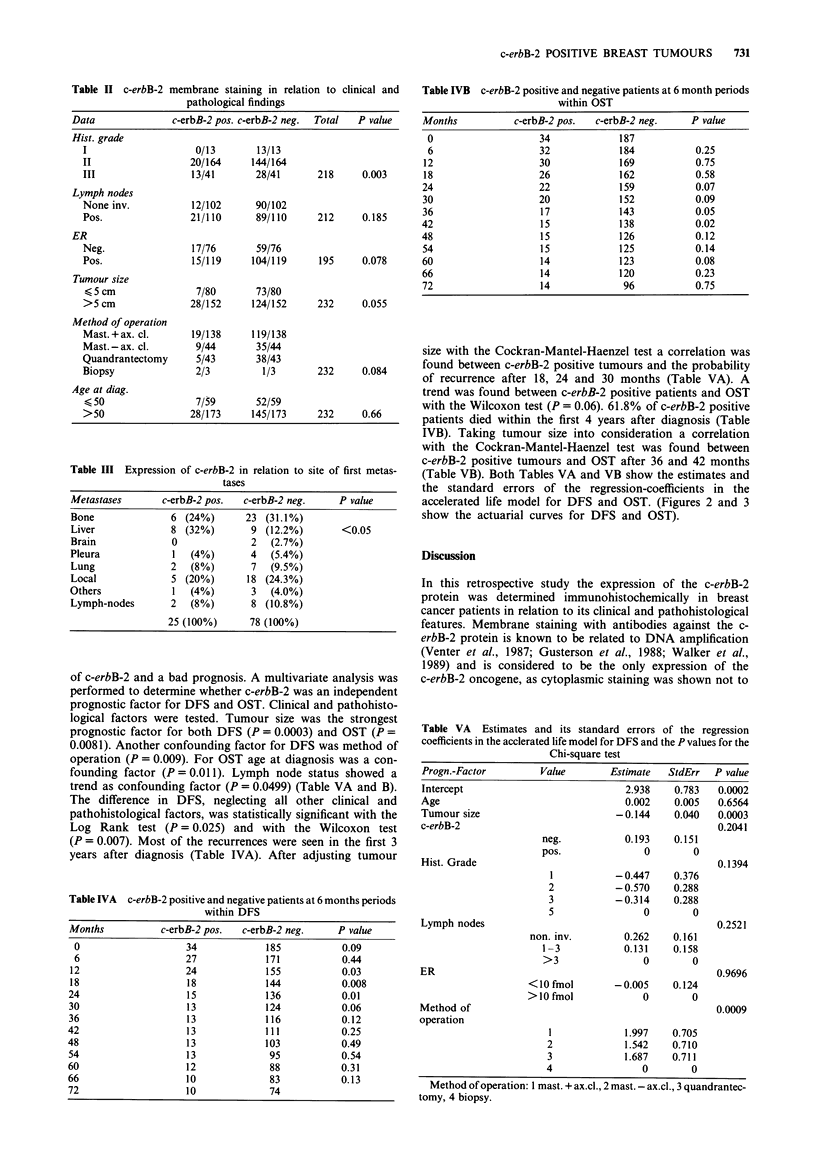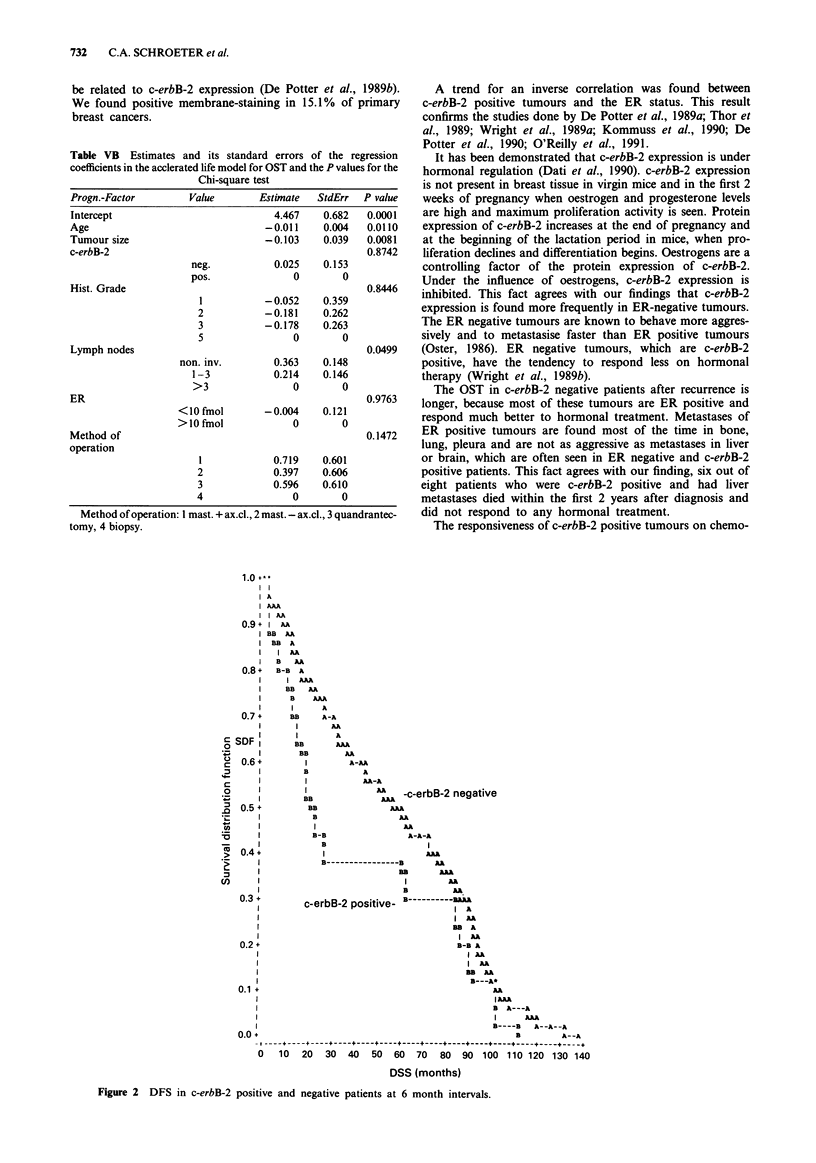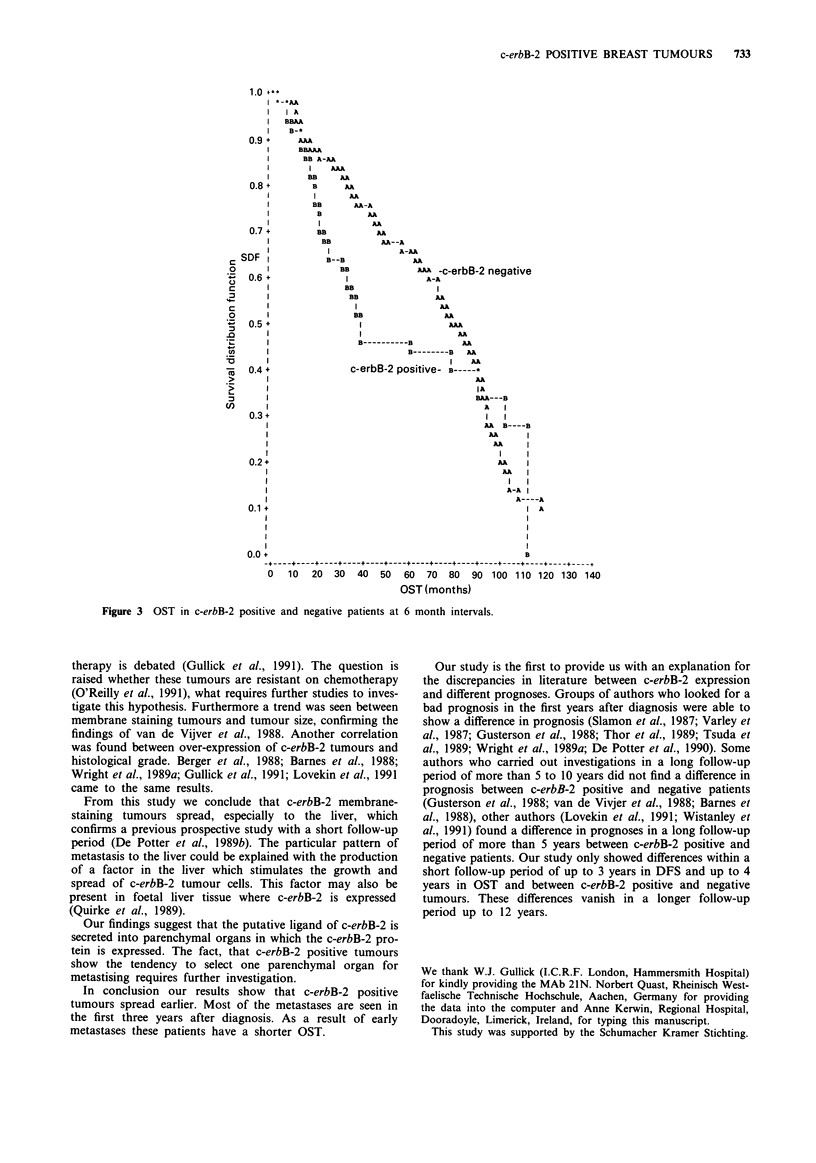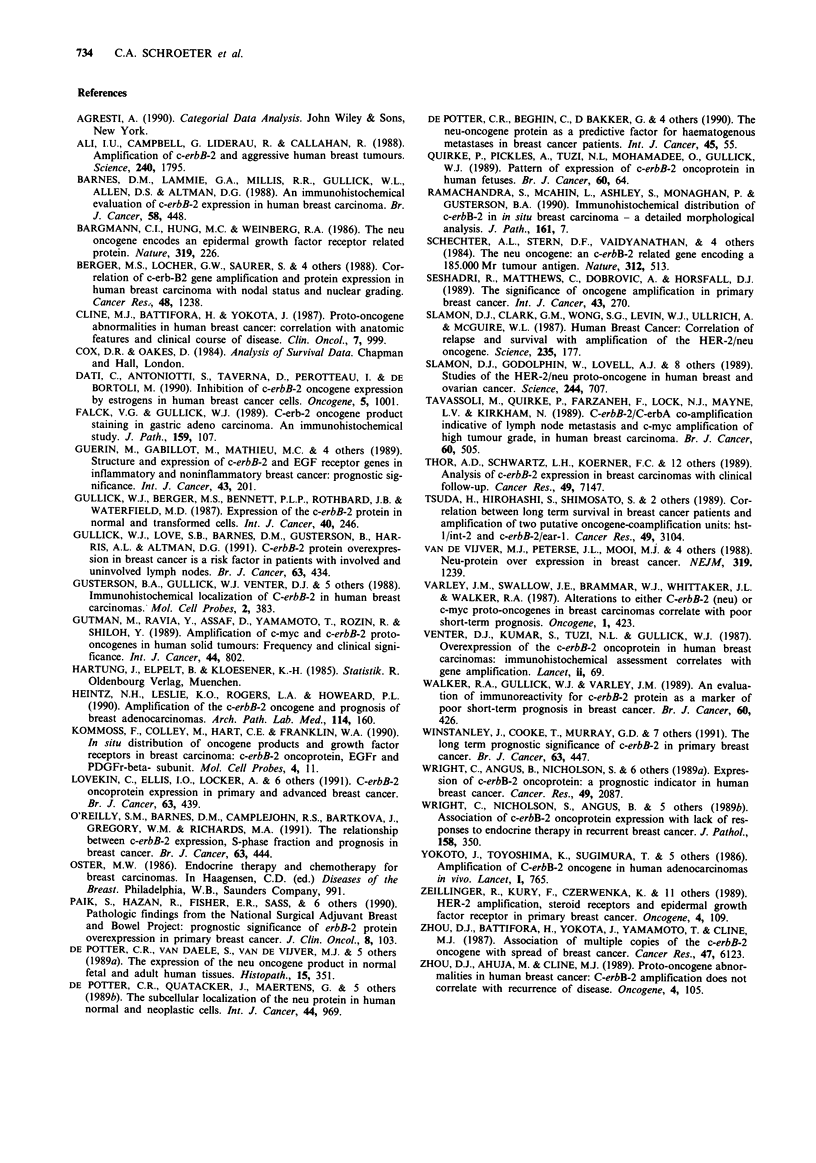# c-erbB-2 positive breast tumours behave more aggressively in the first years after diagnosis.

**DOI:** 10.1038/bjc.1992.347

**Published:** 1992-10

**Authors:** C. A. Schroeter, C. R. De Potter, K. Rathsmann, R. G. Willighagen, J. C. Greep

**Affiliations:** Department of Surgery, University Hospital, Maastricht, The Netherlands.

## Abstract

**Images:**


Br. J. Cancer (1992), 66, 728-734 © Macmillan Press Ltd., 1992

### c-*erb*B-2 positive breast tumours behave more aggressively in the first years after diagnosis

C.A. Schroeter[1], C.R. De Potter[2], K. Rathsmann[3], R.G.J. Willighagen[4] & J.C. Greep[1]

[1]*Department of Surgery, University Hospital, Maastricht, The Netherlands;* [2]*N. Goormaghtigh Institute of Pathology, University Hospital, Gent, Belgium;* [3]*Department of Medical Statistics, Rheinisch Westfaelische Technische Hochschule, Aachen, Germany;* [4]*Department of Pathology, University Hospital, Maastricht, The Netherlands.*

**Summary** In a retrospective study the expression of the c-*erb*B-2 oncogene was determined immunohistochemically in 276 breast cancer samples from 253 patients with the antibody 21N. The follow-up period was between 7 and 12 years. This study showed a trend for an inverse relationship between c-*erb*B-2 positive tumours and estrogen receptors (ER). A correlation was assessed between c-*erb*B-2 positive tumours and histological grade, liver metastases as first site of metastases, disease free survival time (DFS) in the second and third year after diagnosis and overall survival time (OST) in the third and fourth year after diagnosis. A trend was seen between c-*erb*B-2 positive tumours and tumour size. No correlation was found between c-*erb*B-2 positive tumours and age at diagnosis. The method of operation and lymph node involvement. From this study we conclude that there is a significant difference in prognosis the first years after diagnosis, but this difference seems to vanish in a longer follow-up period of 12 years. This provides us with an explanation for the discrepancies in literature concerning c-*erb*B-2 expression and prognosis in breast cancer. Some investigators did not show differences in prognosis between positive and negative cases after a long follow-up period whereas investigations with a short term follow-up period up to 2-3 years have indeed established a more aggressive behaviour of c-*erb*B-2 overexpressionary tumours.

The c-*erb*B-2 or HER-2/neu oncogene encodes a transmembraneous glycoprotein with tyrosine kinase activity. The gene was first described in rat neuroglioblastoma induced by treatment with a carcinogen (Schechter *et al.*, 1984). C-*erb*B-2 or neu has important sequence homology with the epidermal growth factor receptor. (Schechter *et al.*, 1984; Bargmann *et al.*, 1986). Amplification and overexpression were found especially in breast cancer (Yakota *et al.*, 1986) and gastric carcinomas (Falck & Gullick, 1989).

Increased copy numbers in breast carcinomas were related to bad prognosis by some authors (Cline *et al.*, 1987; Slamon *et al.*, 1987; Varley *et al.*, 1987; Slamon *et al.*, 1989). These results were refuted by others (Ali *et al.*, 1988; Zhou *et al.*, 1989). Gene amplification of c-*erbB*-2 correlated with lymph-node involvement (Slamon *et al.*, 1987; Zhou *et al.*, 1987; Slamon *et al.*, 1989; Guerin *et al.*, 1989; Tavassoli *et al.*, 1989), histological grade (Berger *et al.*, 1988; Tavassoli *et al.*, 1989; Tsuda *et al.*, 1989, Paik *et al.*, 1990), negative ER-content (Cline *et al.*, 1987; Guerin *et al.*, 1989; Zeillinger *et al.*, 1989; Heintz *et al.*, 1990), early recurrence (Zhou *et al.*, 1987; Cline *et al.*, 1987; Varley *et al.*, 1987), short overall survival time (cline *et al.*, 1987; Slamon *et al.*, 1987; Varley *et al.*, 1987; Slamon *et al.*, 1989; Paik *et al.*, 1990) and increased mitotic activity (Heintz *et al.*, 1990; Ramachandra *et al.*, 1990). All of these factors are considered to be bad prognostic indicators. According to other authors there was no correlation with tumour size (Gutman *et al.*, 1989; Seshadri *et al.*, 1989) and age at diagnosis (Zhou *et al.*, 1987; Seshadri *et al.*, 1989).

Studies were also carried out on the protein of c-*erb*B-2 by the immunoperoxidase method on primary cancers and metastases. Correlations were found between membrane staining tumours and patho-histological findings such as tumour size (van de Vijver *et al.*, 1988), negative ER-content (De Potter *et al.*, 1989*a*; Thor *et al.*, 1989; Wright *et al.*, 1989*a*; Kommoss *et al.*, 1990; De Potter *et al.*, 1990), lymph node involvement (Berger *et al.*, 1988; Thor *et al.*, 1989), histological grade (berger *et al.*, 1988; Barnes *et al.*, 1988; Wright *et al.*, 1989*a*) and survival time (Thor *et al.*, 1989; Wright *et al.*, 1989*a*).

Furthermore a trend towards worse prognosis was found by others (Barnes *et al.*, 1988; Thor *et al.*, 1989; Walker *et al.*, 1989; Paik *et al.*, 1990; De Potter *et al.*, 1990). This immunohistochemical study with a clinical follow-up of up to 12 years was carried out to investigate a putative relationship between the c-*erb*B-2 oncogene and factors for prognosis. The aim of this study was to establish an explanation for the discrepancies in prognosis in the literature in a large number of patients.

### Materials and methods

### *Patients and treatment*

Tumour specimens were investigated from 251 female and two male patients with primary breast cancer from the De-Wever-Ziekenhuis in Heerlen, the Netherlands. Patients were chosen by haphazard from 1978-1982. Tumour samples were embedded in buffered formalin and could be used for the indirect immunoperoxidase method. Haematoxylin slides of the primary tumours and the metastases were reviewed. Clinical and pathohistological data as well as patient follow-ups were assessed. Table IA shows the number of patients in each category of prognostic variables.

The patients were treated surgically depending on the clinical status at the time of diagnosis. For patients with minimal disease, T1, or tumours of $<2$ cm, a breast-saving quadrantectomy or radical mastectomy was performed. For patients with intermediate disease, T2, tumours of 2-5 cm, not fixed on the skin or chest wall, mastectomy and axillary clearance were carried out. In cases of breast-saving operations with negative lymph nodes the regional lymph nodes were treated with radiotherapy. In patients with positive lymph nodes adjuvant chemotherapy, six cycles with cyclophosphamide, methotrexate and flurouracil (CMF) was administered instead of radiotherapy. After the operation, patients with T2 or T3 tumours and negative lymph nodes, but localisation of the tumour in the upper or lower medial quadrant or in the centre, received radiotherapy. If the lymph nodes were involved adjuvant chemotherapy was added.

Correspondence: C.A. Schroeter, Reg. Surg., University Hospital, 6201 BX, Maastricht, The Netherlands
Received 24 December 1990; and in revised form 20 February 1992.

**Table IA** Prognostic data of patients with invasive ductular carcinomas

| Data | Total | Med-Std. | Dev. |
|---|---|---|---|
| *Age* | | 59.07 | 12.89 |
| 20–50 | 59 | | |
| 51–65 | 106 | | |
| 66–90 | 67 | | |
| *ER* | | | |
| neg. | 76 | | |
| pos. | 119 | | |
| *Hist. grade* | | | |
| I | 13 | | |
| II | 164 | | |
| III | 41 | | |
| *Lymph nodes* | | | |
| None inv. | 102 | | |
| 1–3 | 58 | | |
| >3 | 52 | | |
| *c-erbB-2* | | | |
| Pos. | 35 | | |
| Neg. | 197 | | |
| *Tumour size* | | 3.22 | 1.74 |
| <5 cm | 80 | | |
| >5 cm | 132 | | |

**Table IB** Primary and adjuvant treatments in invasive ductal carcinoma patients

| Data | Total |
|---|---|
| Method of operation | 232 |
| Mastectomy with ax. clearance | 138 |
| Simple mastectomy | 44 |
| Quadrantectomy | 43 |
| Biopsy | 3 |
| Radiotherapy | 68 |
| Radio- and chemotherapy | 7 |
| Radiotherapy and Tamoxifen | 9 |
| Chemohormonal therapy | 32 |
| Adjuvant chemotherapy | 46 |
| Adjuvant Tamoxifen | 31 |
| Ovariectomy | 1 |

Paget's disease of the nipple was treated with mastectomy and axillary clearance because of its central position. In patients with T4 tumours a biopsy was taken from the primary tumour to determine the ER-status; courses of chemotherapy were given immediately after diagnosis. Simple mastectomy, radiotherapy or a combination of both followed. From 1980 onward all patients with tumour stage 1–4, postmenopausal and positive ER were given tamoxifen as hormonal treatment.

The general follow-up for breast cancer patients after 1982 was based on clinical status mammography, chest X-ray and laboratory diagnosis. Bone metastases were diagnosed in the skeleton by scintigram, bronchial metastases by chest X-ray and cytology and liver metastases by ultrasound and laboratory investigations.

Local recurrences were pathohistologically confirmed and were treated surgically or with radiotherapy or both or with hormonal adjuvants regardless of the stage of the tumour.

To patients with liver metastases six cycles of CMF were administered. Bone metastases were radiated. If ER was positive, these patients were given Tamoxifen. Lung metastases were treated with six cycles of CMF and with Tamoxifen if ER was positive. Pleural metastases and pleural effusion were treated with an intra-pleural administration of neomycin. Solitary brain metastases were enucleated depending on the localisation. Table IB gives the number of patients with the method of primary and adjuvant treatment. The follow-up period varied between 7 and 12 years, depending on the age of the patients, if they were older than 80 the patients were in part reviewed by their GPs. Some of the patients were not available because of lost follow-up.

### *Indirect immunoperoxidase method*

Five micro m sections from blocks of breast cancer fixed in 4% formalin (buffered with phosphate) and embedded in paraffin were dewaxed, rehydrated and washed in phosphate buffered saline (PBS). The peroxidase-anti-peroxidase technique was applied as follows:

(1) immersion of deparaffinised sections in methanol containing 0.03% hydrogen peroxidase for 20 min to block the endogenous peroxidase activities and incubation with 5% bovine serum albumin for 30 min.
(2) Incubation with rabbit polyclonal anti-c-*erb*B-2 antibody 21N diluted at 1/200 for 60 min (Gullick, ICRF, Hammersmith Hospital, London); and rinsed three times with P.B.S. and 1% bovine serum albumin (B.S.A) for 5 min.
(3) Biotinilated swine-anti-rabbit immunoglobulin diluted at 1/80 for 30 min (Dako-patts, Glostrup – Denmark); and rinsed three times with B.S.A. for 5 min.
(4) Avidin-biotin peroxidase complex for 30 min (Dakopatts, Glostrup – Denmark).
(5) The peroxidase reaction was developed using 3–3 diaminobenzidine (Sigma, St Louis – USA) with 0.01% hydrogen peroxide for 10 min followed by washing in tap water. The nuclei were counterstained with haematoxylin. All sections were dehydrated and mounted. Control specimens were prepared by omitting the primary antibody. One slide identified as being positive for c-*erb*B-2 was taken as positive control.

### *Antibody*

21N as a polyclonal antibody was raised against a synthetic peptide derived from the c-*erb*B-2 oncogene product containing the amino acid residues 1243–1255 of the c-terminus of the protein of c-*erb*B-2 (Gullick *et al.*, 1987).

### *Oestrogen receptor content*

The ER-content was determined in 226 tumour specimens by the dextran coated charcoal technique. The hormonal contents were expressed as fmol $mg^{-1}$ protein. Values of more than 10 fmol $mg^{-1}$ protein were considered as positive, whereas values lower than 10 fmol $mg^{-1}$ protein as ER negative.

### *Pathological assessment*

The slides were reexamined for correct grading and categorising. The size of the tumour and the number of lymph nodes were determined. The breast cancers were divided pathohistologically into 239 invasive ductular carcinomas, 16 intraductular carcinomas (DCIS), 21 invasive lobular carcinomas and two cases of Paget's disease of the nipple. The invasive ductular carcinomas were again divided into stage 1, 2 and 3 according to histological grade.

All primary and secondary tumour specimens were examined by two independent observers. The immunohistochemical staining was scored as positive if there was membrane staining. Cytoplasmic staining was not considered specific for the c-*erb*B-2 protein since only membrane staining was considered specific as previously shown (De Potter *et al.*, 1989*b*).

### *Statistical analysis*

Clinical and pathohistological factors in relation to c-*erb*B-2 over-expression were assessed by Fisher's exact test (Hartung, 1985). Age at diagnosis was calculated with the Wilcoxon Rank Sum test (Hartung, 1985).

In a multivariate analysis, using an accelerated life model (Cox & Oakes, 1984), the relation between DFS and OST and the following prognostic factors as: C-*erb*B-2 over-expression, histology, lymph node status, ER, method of operation, age at diagnosis and tumour size were calculated.

The actuarial curves for DFS and OST were calculated with the Kaplan-Meier technique. The tumour size was the strongest prognostic factor in the accelerated life model. Adjusting for tumour size the probability of recurrence at fixed time periods after 6, 12, 18, . . . 36 months, respectively the probability of survival at fixed time periods after 6, 12, 18, . . . 36 months was assessed under consideration of the c-*erb*B-2 over-expression with the Cockran-Mantel-Haenzel test (Agresti, 1990). Neglecting all other clinical and patho-histological factors DFS and OST were tested for the c-*erb*B-2 over-expression with the Log Rank and Wilcoxon test. All *P*-values are two sided.

### Results

Tumours were only scored as positive if the membrane was stained (Figure 1). A different cytoplasmic staining pattern was observed in some normal cells and some tumour cells, but was not considered specific for the c-*erb*B-2 protein. Each tumour was assessed according to the following criteria:

(1) Scoring of the membrane.
(2) Assessment of different components within the tumour (e.g. invasive duct./intraductular).
(3) Comparison of the staining of primary tumours and involved lymph nodes.

Normal breast tissue, if found, only showed granular cytoplasmic staining. Smooth muscle cells and the upper layers of the epidermis tended to have cytoplasmic staining, which again was not considered specific for the expression of the protein of the c-*erb*B-2 oncogene.

For the statistical analysis only patients with invasive ductular carcinomas were used. Thirty-five of 232 (15.1%) patients with invasive ductular carcinomas showed membrane staining tumours. The median age of the c-*erb*B-2 positive patients was 57.8 and the median age of the negative patients was 59.3.

A trend for an inverse correlation between membrane staining tumours and ER content was seen ($P = 0.078$) (Table II). A correlation between membrane staining tumours and the histological grade was found ($P = 0.003$) (Table II). None of the twenty-one invasive lobular carcinomas was positive for c-*erbB*-2. Three of the four intraductular carcinomas were positive, one of these three also had an an intralobular component, which was negative. An invasive carcinoma grade 3 with an *in situ* component showed membrane staining in the invasive as well as in the *in situ* part. The two cases of Paget's disease of the nipple showed membrane staining and the two underlying invasive grade 2 carcinomas. The Paget cells were of large size with large nuclei and prominent nucleoli.

Concerning the tumour size a trend was found between c-*erb*B-2 tumours and a tumour size larger than 5 cm ($P = 0.055$) (Table II). A correlation was seen between the tumours which expressed the protein of c-*erb*B-2 and site of first metastasis. The liver was the only tissue to have a correlation with c-*erb*B-2 positive tumours ($P < 0.05$) (Table III). No correlation was found between the over-expression of the c-*erb*B-2 oncogen and age at diagnosis ($P = 0.66$), method of operation ($P = 0.084$) and axillary lymph-nodes ($P = 0.18$) (Table II). There was some difference in the membrane staining between primary tumours and their lymph node metastases. In 3/7 (42.9%) of the lymph node metastases the tumour cells showed a less marked membrane staining. The number of positive cells was also lower than in the primary tumour.

There was a significant correlation between over-expression

**Figure 1** Immunohistochemical staining with 21N. Invasive duct-cell carcinoma stained for the c-*erb*B-2 oncogene product with 21N. All tumour cells show membrane staining.

**Table II** c-*erb*B-2 membrane staining in relation to clinical and pathological findings

| *Data* | c-erb*B-2 pos.* | c-erb*B-2 neg.* | *Total* | *P value* |
|---|---|---|---|---|
| *Hist. grade* | | | | |
| I | 0/13 | 13/13 | | |
| II | 20/164 | 144/164 | | |
| III | 13/41 | 28/41 | 218 | 0.003 |
| *Lymph nodes* | | | | |
| None inv. | 12/102 | 90/102 | | |
| Pos. | 21/110 | 89/110 | 212 | 0.185 |
| *ER* | | | | |
| Neg. | 17/76 | 59/76 | | |
| Pos. | 15/119 | 104/119 | 195 | 0.078 |
| *Tumour size* | | | | |
| ≤5 cm | 7/80 | 73/80 | | |
| >5 cm | 28/152 | 124/152 | 232 | 0.055 |
| *Method of operation* | | | | |
| Mast. + ax. cl. | 19/138 | 119/138 | | |
| Mast. – ax. cl. | 9/44 | 35/44 | | |
| Quandrantectomy | 5/43 | 38/43 | | |
| Biopsy | 2/3 | 1/3 | 232 | 0.084 |
| *Age at diag.* | | | | |
| ≤50 | 7/59 | 52/59 | | |
| >50 | 28/173 | 145/173 | 232 | 0.66 |

**Table III** Expression of c-*erb*B-2 in relation to site of first metastases

| *Metastases* | c-erb*B-2 pos.* | c-erb*B-2 neg.* | *P value* |
|---|---|---|---|
| Bone | 6 (24%) | 23 (31.1%) | |
| Liver | 8 (32%) | 9 (12.2%) | <0.05 |
| Brain | 0 | 2 (2.7%) | |
| Pleura | 1 (4%) | 4 (5.4%) | |
| Lung | 2 (8%) | 7 (9.5%) | |
| Local | 5 (20%) | 18 (24.3%) | |
| Others | 1 (4%) | 3 (4.0%) | |
| Lymph-nodes | 2 (8%) | 8 (10.8%) | |
| | 25 (100%) | 78 (100%) | |

of c-*erb*B-2 and a bad prognosis. A multivariate analysis was performed to determine whether c-*erb*B-2 was an independent prognostic factor for DFS and OST. Clinical and pathohistological factors were tested. Tumour size was the strongest prognostic factor for both DFS ($P = 0.0003$) and OST ($P = 0.0081$). Another confounding factor for DFS was method of operation ($P = 0.009$). For OST age at diagnosis was a confounding factor ($P = 0.011$). Lymph node status showed a trend as confounding factor ($P = 0.0499$) (Table VA and B). The difference in DFS, neglecting all other clinical and pathohistological factors, was statistically significant with the Log Rank test ($P = 0.025$) and with the Wilcoxon test ($P = 0.007$). Most of the recurrences were seen in the first 3 years after diagnosis (Table IVA). After adjusting tumour size with the Cockran-Mantel-Haenzel test a correlation was found between c-*erb*B-2 positive tumours and the probability of recurrence after 18, 24 and 30 months (Table VA). A trend was found between c-*erbB*-2 positive patients and OST with the Wilcoxon test ($P = 0.06$). 61.8% of c-*erb*B-2 positive patients died within the first 4 years after diagnosis (Table IVB). Taking tumour size into consideration a correlation with the Cockran-Mantel-Haenzel test was found between c-*erb*B-2 positive tumours and OST after 36 and 42 months (Table VB). Both Tables VA and VB show the estimates and the standard errors of the regression-coefficients in the accelerated life model for DFS and OST. (Figures 2 and 3 show the actuarial curves for DFS and OST).

**Table IVA** c-*erb*B-2 positive and negative patients at 6 months periods within DFS

| *Months* | c-erb*B-2 pos.* | c-erb*B-2 neg.* | *P value* |
|---|---|---|---|
| 0 | 34 | 185 | 0.09 |
| 6 | 27 | 171 | 0.44 |
| 12 | 24 | 155 | 0.03 |
| 18 | 18 | 144 | 0.008 |
| 24 | 15 | 136 | 0.01 |
| 30 | 13 | 124 | 0.06 |
| 36 | 13 | 116 | 0.12 |
| 42 | 13 | 111 | 0.25 |
| 48 | 13 | 103 | 0.49 |
| 54 | 13 | 95 | 0.54 |
| 60 | 12 | 88 | 0.31 |
| 66 | 10 | 83 | 0.13 |
| 72 | 10 | 74 | |

**Table IVB** c-*erb*B-2 positive and negative patients at 6 month periods within OST

| *Months* | c-erb*B-2 pos.* | c-erb*B-2 neg.* | *P value* |
|---|---|---|---|
| 0 | 34 | 187 | |
| 6 | 32 | 184 | 0.25 |
| 12 | 30 | 169 | 0.75 |
| 18 | 26 | 162 | 0.58 |
| 24 | 22 | 159 | 0.07 |
| 30 | 20 | 152 | 0.09 |
| 36 | 17 | 143 | 0.05 |
| 42 | 15 | 138 | 0.02 |
| 48 | 15 | 126 | 0.12 |
| 54 | 15 | 125 | 0.14 |
| 60 | 14 | 123 | 0.08 |
| 66 | 14 | 120 | 0.23 |
| 72 | 14 | 96 | 0.75 |

### Discussion

In this retrospective study the expression of the c-*erb*B-2 protein was determined immunohistochemically in breast cancer patients in relation to its clinical and pathohistological features. Membrane staining with antibodies against the c-*erb*B-2 protein is known to be related to DNA amplification (Venter *et al.*, 1987; Gusterson *et al.*, 1988; Walker *et al.*, 1989) and is considered to be the only expression of the c-*erb*B-2 oncogene, as cytoplasmic staining was shown not to

**Table VA** Estimates and its standard errors of the regression coefficients in the acclerated life model for DFS and the *P* values for the Chi-square test

| *Progn.-Factor* | *Value* | *Estimate* | *StdErr* | *P value* |
|---|---|---|---|---|
| Intercept | | 2.938 | 0.783 | 0.0002 |
| Age | | 0.002 | 0.005 | 0.6564 |
| Tumour size | | –0.144 | 0.040 | 0.0003 |
| c-*erb*B-2 | | | | 0.2041 |
| | neg. | 0.193 | 0.151 | |
| | pos. | 0 | 0 | |
| Hist. Grade | | | | 0.1394 |
| | 1 | –0.447 | 0.376 | |
| | 2 | –0.570 | 0.288 | |
| | 3 | –0.314 | 0.288 | |
| | 5 | 0 | 0 | |
| Lymph nodes | | | | 0.2521 |
| | non. inv. | 0.262 | 0.161 | |
| | 1–3 | 0.131 | 0.158 | |
| | >3 | 0 | 0 | |
| ER | | | | 0.9696 |
| | <10 fmol | –0.005 | 0.124 | |
| | >10 fmol | 0 | 0 | |
| Method of operation | | | | 0.0009 |
| | 1 | 1.997 | 0.705 | |
| | 2 | 1.542 | 0.710 | |
| | 3 | 1.687 | 0.711 | |
| | 4 | 0 | 0 | |

Method of operation: 1 mast. + ax.cl., 2 mast. – ax.cl., 3 quandrantectomy, 4 biopsy.

be related to c-*erb*B-2 expression (De Potter *et al.*, 1989*b*). We found positive membrane-staining in 15.1% of primary breast cancers.

**Table VB** Estimates and its standard errors of the regression coefficients in the acclerated life model for OST and the *P* values for the Chi-square test

| *Progn.-Factor* | *Value* | *Estimate* | *StdErr* | *P value* |
|---|---|---|---|---|
| Intercept | | 4.467 | 0.682 | 0.0001 |
| Age | | −0.011 | 0.004 | 0.0110 |
| Tumour size | | −0.103 | 0.039 | 0.0081 |
| c-*erb*B-2 | | | | 0.8742 |
| | neg. | 0.025 | 0.153 | |
| | pos. | 0 | 0 | |
| Hist. Grade | | | | 0.8446 |
| | 1 | −0.052 | 0.359 | |
| | 2 | −0.181 | 0.262 | |
| | 3 | −0.178 | 0.263 | |
| | 5 | 0 | 0 | |
| Lymph nodes | | | | 0.0499 |
| | non. inv. | 0.363 | 0.148 | |
| | 1–3 | 0.214 | 0.146 | |
| | >3 | 0 | 0 | |
| ER | | | | 0.9763 |
| | <10 fmol | −0.004 | 0.121 | |
| | >10 fmol | 0 | 0 | |
| Method of operation | | | | 0.1472 |
| | 1 | 0.719 | 0.601 | |
| | 2 | 0.397 | 0.606 | |
| | 3 | 0.596 | 0.610 | |
| | 4 | 0 | 0 | |

Method of operation: 1 mast. + ax.cl., 2 mast. − ax.cl., 3 quandrantectomy, 4 biopsy.

A trend for an inverse correlation was found between c-*erb*B-2 positive tumours and the ER status. This result confirms the studies done by De Potter *et al.*, 1989*a*; Thor *et al.*, 1989; Wright *et al.*, 1989*a*; Kommuss *et al.*, 1990; De Potter *et al.*, 1990; O'Reilly *et al.*, 1991.

It has been demonstrated that c-*erb*B-2 expression is under hormonal regulation (Dati *et al.*, 1990). c-*erb*B-2 expression is not present in breast tissue in virgin mice and in the first 2 weeks of pregnancy when oestrogen and progesterone levels are high and maximum proliferation activity is seen. Protein expression of c-*erb*B-2 increases at the end of pregnancy and at the beginning of the lactation period in mice, when proliferation declines and differentiation begins. Oestrogens are a controlling factor of the protein expression of c-*erb*B-2. Under the influence of oestrogens, c-*erb*B-2 expression is inhibited. This fact agrees with our findings that c-*erb*B-2 expression is found more frequently in ER-negative tumours. The ER negative tumours are known to behave more aggressively and to metastasise faster than ER positive tumours (Oster, 1986). ER negative tumours, which are c-*erb*B-2 positive, have the tendency to respond less on hormonal therapy (Wright *et al.*, 1989*b*).

The OST in c-*erb*B-2 negative patients after recurrence is longer, because most of these tumours are ER positive and respond much better to hormonal treatment. Metastases of ER positive tumours are found most of the time in bone, lung, pleura and are not as aggressive as metastases in liver or brain, which are often seen in ER negative and c-*erb*B-2 positive patients. This fact agrees with our finding, six out of eight patients who were c-*erb*B-2 positive and had liver metastases died within the first 2 years after diagnosis and did not respond to any hormonal treatment.

The responsiveness of c-*erb*B-2 positive tumours on chemo-

**Figure 2** DFS in c-*erb*B-2 positive and negative patients at 6 month intervals.

**Figure 3** OST in c-*erb*B-2 positive and negative patients at 6 month intervals.

therapy is debated (Gullick *et al.*, 1991). The question is raised whether these tumours are resistant on chemotherapy (O'Reilly *et al.*, 1991), what requires further studies to investigate this hypothesis. Furthermore a trend was seen between membrane staining tumours and tumour size, confirming the findings of van de Vijver *et al.*, 1988. Another correlation was found between over-expression of c-*erb*B-2 tumours and histological grade. Berger *et al.*, 1988; Barnes *et al.*, 1988; Wright *et al.*, 1989*a*; Gullick *et al.*, 1991; Lovekin *et al.*, 1991 came to the same results.

From this study we conclude that c-*erb*B-2 membrane-staining tumours spread, especially to the liver, which confirms a previous prospective study with a short follow-up period (De Potter *et al.*, 1989*b*). The particular pattern of metastasis to the liver could be explained with the production of a factor in the liver which stimulates the growth and spread of c-*erb*B-2 tumour cells. This factor may also be present in foetal liver tissue where c-*erb*B-2 is expressed (Quirke *et al.*, 1989).

Our findings suggest that the putative ligand of c-*erb*B-2 is secreted into parenchymal organs in which the c-*erb*B-2 protein is expressed. The fact, that c-*erb*B-2 positive tumours show the tendency to select one parenchymal organ for metastising requires further investigation.

In conclusion our results show that c-*erb*B-2 positive tumours spread earlier. Most of the metastases are seen in the first three years after diagnosis. As a result of early metastases these patients have a shorter OST.

Our study is the first to provide us with an explanation for the discrepancies in literature between c-*erb*B-2 expression and different prognoses. Groups of authors who looked for a bad prognosis in the first years after diagnosis were able to show a difference in prognosis (Slamon *et al.*, 1987; Varley *et al.*, 1987; Gusterson *et al.*, 1988; Thor *et al.*, 1989; Tsuda *et al.*, 1989; Wright *et al.*, 1989*a*; De Potter *et al.*, 1990). Some authors who carried out investigations in a long follow-up period of more than 5 to 10 years did not find a difference in prognosis between c-*erbB*-2 positive and negative patients (Gusterson *et al.*, 1988; van de Vivjer *et al.*, 1988; Barnes *et al.*, 1988), other authors (Lovekin *et al.*, 1991; Wistanley *et al.*, 1991) found a difference in prognoses in a long follow-up period of more than 5 years between c-*erb*B-2 positive and negative patients. Our study only showed differences within a short follow-up period of up to 3 years in DFS and up to 4 years in OST and between c-*erb*B-2 positive and negative tumours. These differences vanish in a longer follow-up period up to 12 years.

We thank W.J. Gullick (I.C.R.F. London, Hammersmith Hospital) for kindly providing the MAb 21N. Norbert Quast, Rheinisch Westfaelische Technische Hochschule, Aachen, Germany for providing the data into the computer and Anne Kerwin, Regional Hospital, Dooradoyle, Limerick, Ireland, for typing this manuscript.

This study was supported by the Schumacher Kramer Stichting.

### References

AGRESTI, A. (1990). *Categorial Data Analysis*. John Wiley & Sons, New York.

ALI, I.U., CAMPBELL, G. LIDERAU, R. & CALLAHAN, R. (1988). Amplification of c-*erbB*-2 and aggressive human breast tumours. *Science*, **240**, 1795.

BARNES, D.M., LAMMIE, G.A., MILLIS, R.R., GULLICK, W.L., ALLEN, D.S. & ALTMAN, D.G. (1988). An immunohistochemical evaluation of c-*erbB*-2 expression in human breast carcinoma. *Br. J. Cancer*, **58**, 448.

BARGMANN, C.I., HUNG, M.C. & WEINBERG, R.A. (1986). The neu oncogene encodes an epidermal growth factor receptor related protein. *Nature*, **319**, 226.

BERGER, M.S., LOCHER, G.W., SAURER, S. & 4 others (1988). Correlation of c-erb-B2 gene amplification and protein expression in human breast carcinoma with nodal status and nuclear grading. *Cancer Res.*, **48**, 1238.

CLINE, M.J., BATTIFORA, H. & YOKOTA, J. (1987). Proto-oncogene abnormalities in human breast cancer: correlation with anatomic features and clinical course of disease. *Clin. Oncol.*, **7**, 999.

COX, D.R. & OAKES, D. (1984). *Analysis of Survival Data*. Chapman and Hall, London.

DATI, C., ANTONIOTTI, S., TAVERNA, D., PEROTTEAU, I. & DE BORTOLI, M. (1990). Inhibition of c-*erbB*-2 oncogene expression by estrogens in human breast cancer cells. *Oncogene*, **5**, 1001.

FALCK, V.G. & GULLICK, W.J. (1989). C-erb-2 oncogene product staining in gastric adeno carcinoma. An immunohistochemical study. *J. Path.*, **159**, 107.

GUERIN, M., GABILLOT, M., MATHIEU, M.C. & 4 others (1989). Structure and expression of c-*erbB*-2 and EGF receptor genes in inflammatory and noninflammatory breast cancer: prognostic significance. *Int. J. Cancer*, **43**, 201.

GULLICK, W.J., BERGER, M.S., BENNETT, P.L.P., ROTHBARD, J.B. & WATERFIELD, M.D. (1987). Expression of the c-*erbB*-2 protein in normal and transformed cells. *Int. J. Cancer*, **40**, 246.

GULLICK, W.J., LOVE, S.B., BARNES, D.M., GUSTERSON, B., HARRIS, A.L. & ALTMAN, D.G. (1991). C-*erbB*-2 protein overexpression in breast cancer is a risk factor in patients with involved and uninvolved lymph nodes. *Br. J. Cancer*, **63**, 434.

GUSTERSON, B.A., GULLICK, W.J. VENTER, D.J. & 5 others (1988). Immunohistochemical localization of C-*erbB*-2 in human breast carcinomas. *Mol. Cell Probes*, **2**, 383.

GUTMAN, M., RAVIA, Y., ASSAF, D., YAMAMOTO, T., ROZIN, R. & SHILOH, Y. (1989). Amplification of c-myc and c-*erbB*-2 proto-oncogenes in human solid tumours: Frequency and clinical significance. *Int. J. Cancer*, **44**, 802.

HARTUNG, J., ELPELT, B. & KLOESENER, K.-H. (1985). *Statistik*. R. Oldenbourg Verlag, Muenchen.

HEINTZ, N.H., LESLIE, K.O., ROGERS, L.A. & HOWEARD, P.L. (1990). Amplification of the c-*erbB*-2 oncogene and prognosis of breast adenocarcinomas. *Arch. Path. Lab. Med.*, **114**, 160.

KOMMOSS, F., COLLEY, M., HART, C.E. & FRANKLIN, W.A. (1990). *In situ* distribution of oncogene products and growth factor receptors in breast carcinoma: c-*erbB*-2 oncoprotein, EGFr and PDGFr-beta- subunit. *Mol. Cell Probes*, **4**, 11.

LOVEKIN, C., ELLIS, I.O., LOCKER, A. & 6 others (1991). C-*erbB*-2 oncoprotein expression in primary and advanced breast cancer. *Br. J. Cancer*, **63**, 439.

O'REILLY, S.M., BARNES, D.M., CAMPLEJOHN, R.S., BARTKOVA, J., GREGORY, W.M. & RICHARDS, M.A. (1991). The relationship between c-*erbB*-2 expression, S-phase fraction and prognosis in breast cancer. *Br. J. Cancer*, **63**, 444.

OSTER, M.W. (1986). Endocrine therapy and chemotherapy for breast carcinomas. In Haagensen, C.D. (ed.) *Diseases of the Breast*. Philadelphia, W.B., Saunders Company, 991.

PAIK, S., HAZAN, R., FISHER, E.R., SASS, & 6 others (1990). Pathologic findings from the National Surgical Adjuvant Breast and Bowel Project: prognostic significance of *erbB*-2 protein overexpression in primary breast cancer. *J. Clin. Oncol.*, **8**, 103.

DE POTTER, C.R., VAN DAELE, S., VAN DE VIJVER, M.J. & 5 others (1989*a*). The expression of the neu oncogene product in normal fetal and adult human tissues. *Histopath.*, **15**, 351.

DE POTTER, C.R., QUATACKER, J., MAERTENS, G. & 5 others (1989*b*). The subcellular localization of the neu protein in human normal and neoplastic cells. *Int. J. Cancer*, **44**, 969.

DE POTTER, C.R., BEGHIN, C., D BAKKER, G. & 4 others (1990). The neu-oncogene protein as a predictive factor for haematogenous metastases in breast cancer patients. *Int. J. Cancer*, **45**, 55.

QUIRKE, P., PICKLES, A., TUZI, N.L, MOHAMADEE, O., GULLICK, W.J. (1989). Pattern of expression of c-*erbB*-2 oncoprotein in human fetuses. *Br. J. Cancer*, **60**, 64.

RAMACHANDRA, S., MCAHIN, L., ASHLEY, S., MONAGHAN, P. & GUSTERSON, B.A. (1990). Immunohistochemical distribution of c-*erb*B-2 in *in situ* breast carcinoma – a detailed morphological analysis. *J. Path.*, **161**, 7.

SCHECHTER, A.L., STERN, D.F., VAIDYANATHAN, & 4 others (1984). The neu oncogene: an c-*erb*B-2 related gene encoding a 185.000 Mr tumour antigen. *Nature*, **312**, 513.

SESHADRI, R., MATTHEWS, C., DOBROVIC, A. & HORSFALL, D.J. (1989). The significance of oncogene amplification in primary breast cancer. *Int. J. Cancer*, **43**, 270.

SLAMON, D.J., CLARK, G.M., WONG, S.G., LEVIN, W.J., ULLRICH, A. & MCGUIRE, W.L. (1987). Human Breast Cancer: Correlation of relapse and survival with amplification of the HER-2/neu oncogene. *Science*, **235**, 177.

SLAMON, D.J., GODOLPHIN, W., LOVELL, A.J. & 8 others (1989). Studies of the HER-2/neu proto-oncogene in human breast and ovarian cancer. *Science*, **244**, 707.

TAVASSOLI, M., QUIRKE, P., FARZANEH, F., LOCK, N.J., MAYNE, L.V. & KIRKHAM, N. (1989). *C-erbB*-2/C-erbA co-amplification indicative of lymph node metastasis and c-myc amplification of high tumour grade, in human breast carcinoma. *Br. J. Cancer*, **60**, 505.

THOR, A.D., SCHWARTZ, L.H., KOERNER, F.C. & 12 others (1989). Analysis of c-*erbB*-2 expression in breast carcinomas with clinical follow-up. *Cancer Res.*, **49**, 7147.

TSUDA, H., HIROHASHI, S., SHIMOSATO, S. & 2 others (1989). Correlation between long term survival in breast cancer patients and amplification of two putative oncogene-coamplification units: hst-1/int-2 and c-*erbB*-2/ear-1. *Cancer Res.*, **49**, 3104.

VAN DE VIJVER, M.J., PETERSE, J.L., MOOI, M.J. & 4 others (1988). Neu-protein over expression in breast cancer. *NEJM*, **319**. 1239.

VARLEY, J.M., SWALLOW, J.E., BRAMMAR, W.J., WHITTAKER, J.L. & WALKER, R.A. (1987). Alterations to either C-*erbB*-2 (neu) or c-myc proto-oncogenes in breast carcinomas correlate with poor short-term prognosis. *Oncogene*, **1**, 423.

VENTER, D.J., KUMAR, S., TUZI, N.L. & GULLICK, W.J. (1987). Overexpression of the c-*erbB*-2 oncoprotein in human breast carcinomas: immunohistochemical assessment correlates with gene amplification. *Lancet*, **ii**, 69.

WALKER, R.A., GULLICK, W.J. & VARLEY, J.M. (1989). An evaluation of immunoreactivity for c-*erbB*-2 protein as a marker of poor short-term prognosis in breast cancer. *Br. J. Cancer*, **60**, 426.

WINSTANLEY, J., COOKE, T., MURRAY, G.D. & 7 others (1991). The long term prognostic significance of c-*erbB*-2 in primary breast cancer. *Br. J. Cancer*, **63**, 447.

WRIGHT, C., ANGUS, B., NICHOLSON, S. & 6 others (1989*a*). Expression of c-*erbB*-2 oncoprotein: a prognostic indicator in human breast cancer. *Cancer. Res.*, **49**, 2087.

WRIGHT, C., NICHOLSON, S., ANGUS, B. & 5 others (1989*b*). Association of c-*erb*B-2 oncoprotein expression with lack of responses to endocrine therapy in recurrent breast cancer. *J. Pathol.*, **158**, 350.

YOKOTO, J., TOYOSHIMA, K., SUGIMURA, T. & 5 others (1986). Amplification of C-*erbB*-2 oncogene in human adenocarcinomas *in vivo*. *Lancet*, **I**, 765.

ZEILLINGER, R., KURY, F., CZERWENKA, K. & 11 others (1989). HER-2 amplification, steroid receptors and epidermal growth factor receptor in primary breast cancer. *Oncogene*, **4**, 109.

ZHOU, D.J., BATTIFORA, H., YOKOTA, J., YAMAMOTO, T. & CLINE, M.J. (1987). Association of multiple copies of the c-*erbB*-2 oncogene with spread of breast cancer. *Cancer Res.*, **47**, 6123.

ZHOU, D.J., AHUJA, M. & CLINE, M.J. (1989). Proto-oncogene abnormalities in human breast cancer: C-*erb*B-2 amplification does not correlate with recurrence of disease. *Oncogene*, **4**, 105.